# An epidemiological study of paediatric motocross injuries in the United Kingdom

**DOI:** 10.1007/s11832-015-0685-5

**Published:** 2015-09-18

**Authors:** Rohit Singh, Akshay Malhotra, Nigel Kyle, Stuart Hay

**Affiliations:** Robert Jones Agnes Hunt Orthopaedic Hospital, Oswestry, UK

**Keywords:** Motocross, Paediatric, Injuries, Cost

## Abstract

**Purpose:**

Although off-road motorcycling is one of the most popular sports activities practised by millions of people worldwide, little has been written on motocross injuries and their prevention. In the UK alone, motocross has grown into a phenomenally ambitious and popular franchise. There are >200 motocross clubs across the country holding >900 events annually. The aim of this study is to categorise and quantify the magnitude of motocross paediatric injuries and associated morbidity.

**Methods:**

Data were collected prospectively over 4 years (2010−2014) at our unit. All injuries caused by motocross biking that were referred to our trauma and orthopaedic department were included in this study, regardless of whether the rider was performing the sport competitively or recreationally.

**Results:**

During the study period, 130 patients (aged 4−17 years) were identified with a total of 142 injuries, ranging from one to six injuries per patient. Most of the injuries were sustained within the early spring and summer months, representing the start of the motocross season; 76 patients required hospital admission, with 60 (42 %) requiring surgical intervention.

**Conclusion:**

We present the first epidemiological study of motocross paediatric injuries in the UK. The results from this study highlight the frequency and severity of motocross-related injuries in the paediatric population in the UK. This may assist in providing recommendations and guidelines to governing bodies and to parents. The injuries sustained during motocross have significant resource implications, especially for smaller rural hospitals, as shown by the number of injuries doubling over the past 4 years.

## Introduction

Although off-road motorcycling is one of the most popular sports activities practised by millions of people worldwide, little has been written on motocross injuries and their prevention. The sport of motocross has been a recreational and competitive sport for >100 years. It is a form of motorcycle racing held on established off-road circuits. It initially evolved from motorcycle trial competitions back in 1906 [1, 2]. Motocross has grown in popularity across the globe, with the franchise becoming more appealing with its publicity and accessibility [1, 2]. The first competitive competition in the UK took place at Camberley, Surrey in 1924 [3]; however, there are now >200 motocross clubs across the country holding >900 annual events [4].

Motocross competitive racing has age categories ranging from 6 years for boys and girls to 65 years for competitive competitions. However, it has been well documented that 3-year-old children have been taking part competitively [5]. The motocross bikes available on the market are extremely powerful and can reach speeds of approximately 100 mph and weigh approximately 115 kg [1, 3, 4].

Motocross race events occur on most weekends throughout the summer, with approximately 100–200 young competitors per race. The season for nationwide competitions is between March and October; however, recreational sport and training continues throughout the year. The sport is readily accessible via online license application with membership fees of one hundred and fifty pounds sterling.

Healthcare professionals at our rural trauma unit have noticed severe injuries in children and teenagers participating in motocross events.

Although the distribution and severity of motocross injuries in the paediatric population are not known, there are plenty of studies in the literature regarding injuries in competitive motorcycle and motorcar racing [6, 7]. Given that no studies have been published to date exploring the relationship between motocross sporting events and the associated injury patterns in children, this study was designed prospectively to review our experience and quantify those observations.

Historically, the majority of paediatric injuries have been managed non-surgically. However, there has been a 10-fold increase over the last two decades of the 20th century in surgical treatment for paediatric fractures [8, 9]. We hypothesise that paediatric injuries sustained during motocross require frequent surgical interventions and the frequency of injuries is increasing. We present the first epidemiological study of paediatric motocross injuries in the UK.

## Method

Data were prospectively collected at our unit over a 4-year period from August 2010 to August 2014. All injuries caused by motocross vehicles that were referred to our trauma and orthopaedic department were included in this study, regardless of whether the rider was performing motocross competitively or recreationally (training). Patients were either seen acutely in the emergency department or subsequently in the fracture clinic.

We specifically only looked at motocross riders. Injuries sustained while off-road mountain biking, or using motorcycles were excluded from our study.

Injuries were identified in 130 patients who were directly referred to our department following recreational or competitive motocross.

The information received included basic details (name, age, and sex), the type of injury sustained, the need for admission/surgery, and any associated complications. The information was prospectively collected form dictated hospital notes (inpatient and outpatient) from trauma and orthopaedic consultants and specialist registrars in our department. Results were tabulated using an excel spreadsheet (Microsoft, Redmond, WA, USA).

To facilitate data handling, the injuries were then classified into 16 categories according to the severity and region of injury.

## Results

During the 4-year study period (August 2010 to August 2014), 130 patients were referred to our trauma and orthopaedic department. A total of 142 injuries were recorded, ranging from one to six injuries per patient. The vast majority of injuries occurred in patients engaged in the sport for competitive purposes as opposed to recreational purposes.

**Fig. 1:**
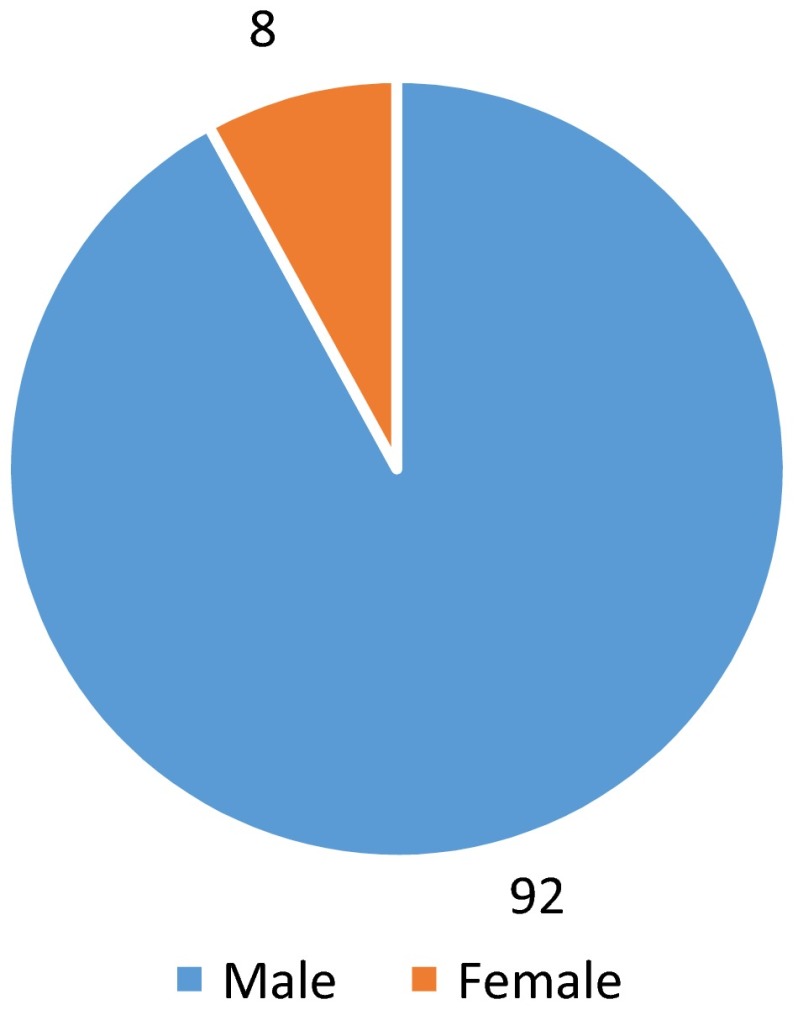
Pie chart showing percentage of motocross injuries according to gender

The vast majority of injuries were sustained in male patients (*n* = 120, 92 %) (Fig. 1).

**Fig. 2:**
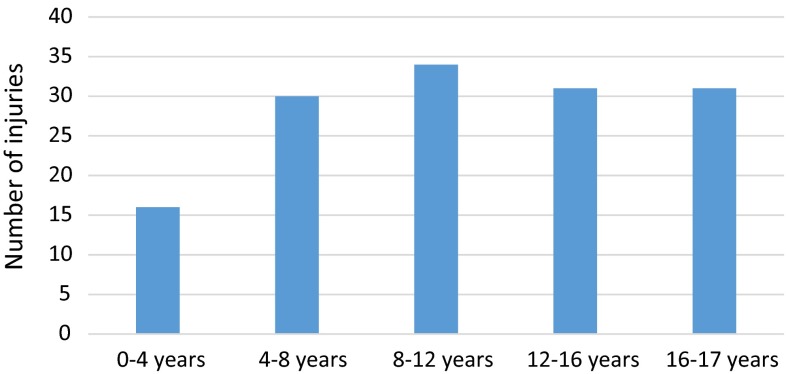
Bar chart showing age distribution of motocross injuries

The age distribution is shown in Fig. 2.

**Fig. 3:**
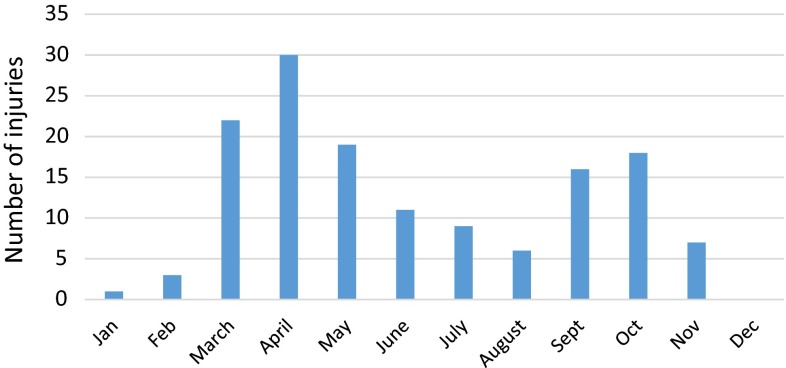
Bar chart showing distribution of motocross injuries according to month

The majority of injuries were sustained within the transitional period from winter to early spring (Fig. 3), which coincides with the start of the motocross season (March), where riders may be more vulnerable after the winter break, i.e., being unaccustomed to the competitive terrain and ‘rusty’ in terms of skill level. There is a noticeable peak again during the transition between autumn and winter where we suspect the course will be more treacherous as the weather conditions become more hazardous. We note that there is a decline in the summer months which can be explained by the optimum ratio between skill level and dry conditions which would suit the racers and cause fewer collisions/accidents.

**Fig. 4:**
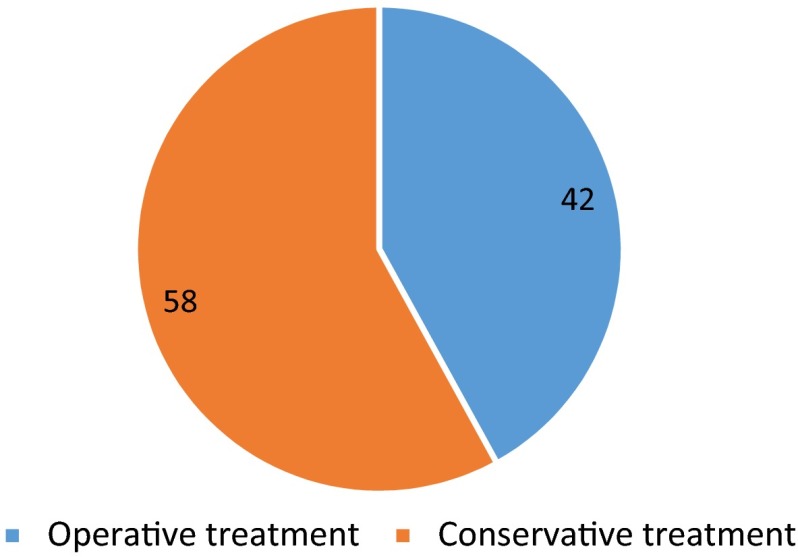
Pie chart showing percentage of motocross patients receiving surgical treatment

A total of 60 (42 %) injuries required surgical treatment (Fig. 4), some requiring multiple procedures (two or more procedures) with a prolonged hospital stay of ≥3 days (*n* = 20, 15 %).

**Fig. 5:**
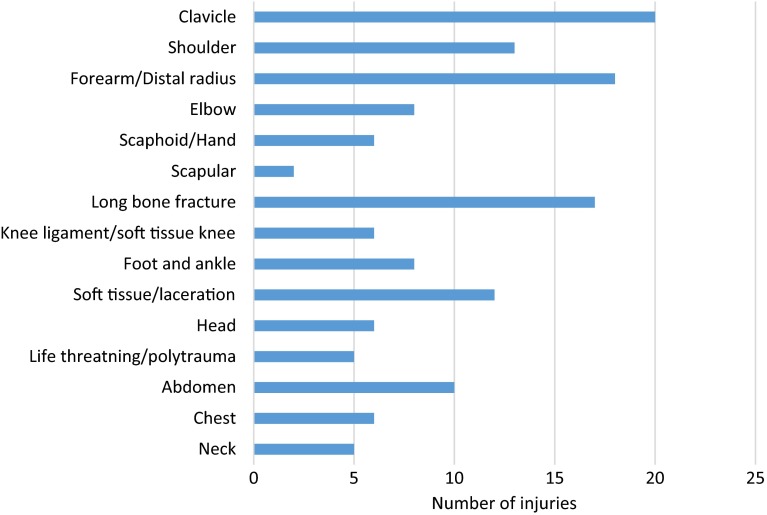
Bar chart showing anatomical distribution of motocross injuries

There was a large variety of injuries ranging from soft tissue lacerations to life-threatening injuries (Fig. 5).

The most common injuries were clavicle fractures (*n* = 20, 14 %), forearm/distal radius fractures (*n* = 18, 13 %) including 10 forearm fractures and 8 distal radius fractures, followed by long bone fractures (femur and tibia) (*n* = 17, 12 %).

A significant proportion of the patients in this series suffered two or more injuries (*n* = 26, 18 %).

A total of 76 (54 %) patients required in-patient hospital stay during the study period. The length of hospital stay ranged from 1–7 days with an average of 2 days. A total of 8 patients required admission to HDU/ITU; 5 of these required emergency laparotomy for intra-abdominal haemorrhage, and three required monitoring following chest injuries.

All patients required formal orthopaedic follow-up in fracture clinics. 34 patients were not residents in the local area of our unit, and follow-up for these patients was arranged at their respective local departments. All local patients required at least two follow-up appointments, regardless of whether they received surgical or non-surgical treatment. To date, the total number of fracture clinic appointments is 550 with an average of four appointments per patient (in our unit).

During the study period there were four serious head injuries which required transfer to neighbouring neurosurgical units for formal decompression and craniotomy. These four patients have been left with neurological complications resulting from intracranial bleeding which resulted in these patients needing full-time care.

It is important to note the occurrence of orthopaedic injuries with long-term implications. Four physeal injuries in the distal femur which are still being closely followed up were all Salter Harris II fractures, with two of these being displaced. The non-displaced fractures were treated with long leg casting and the displaced fractures were treated with closed reduction and percutaneous pinning followed by casting. One of these patients had a small degree of limb length discrepancy. 5 patients sustained soft tissue knee injuries including cruciate ligament and meniscal injuries, which required arthroscopic procedures. Four supracondylar humerus fractures were Gartland type III and were treated with closed reduction and percutaneous pinning, 1 patient had pin migration and two suffered residual stiffness (resolved at 6-month follow-up).

**Fig. 6:**
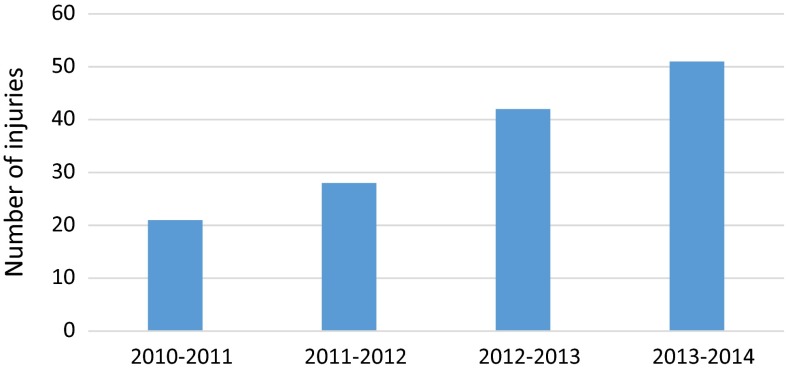
Bar chart showing number of injuries in separate time frames over the last 4 years

During the 4-year study period, we observed a steady rise in the number of injuries (Fig. 6).

We saw 21 injuries in the first year, compared to 51 injuries in the fourth year, showing that the number of injuries more than doubled in the 4-year study period.

**Fig. 7:**
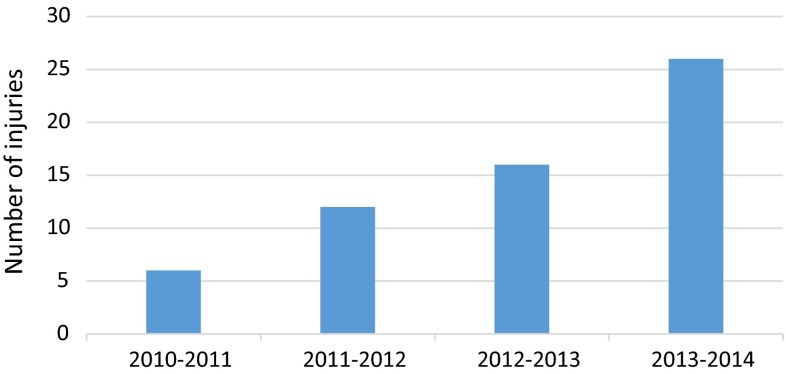
Bar chart showing number of operations per year from motocross injuries

There was also a rise in the number of operations in the treatment of motocross injuries (Fig. 7); only 6 operations were required in the first year compared to 26 in the fourth year, which represents a four-fold increase.

## Discussion

This spectrum of injuries represents the first epidemiological study of this magnitude in the UK for motocross injuries in children.

Although there are many articles in the literature on the patterns of injuries associated with motorcycle accidents and their prevention [10, 11], the patterns and outcomes of injuries associated with motocross accidents in children has not been reported.

Motocross is a popular sport both in the UK and worldwide, with the number of tournaments and racers doubling in the past 5 years [2, 3]. Children as young as 3 years old have participated in this activity. The recognised protective equipment includes kidney belts, neck braces, helmets, goggles, knee and elbow supports; however, despite these, there are still a high number of severe injuries as shown by our data. The injuries sustained in the paediatric population ranged from minor contusions and lacerations to severe life-threatening injuries. Motocross competitions can cause a significant burden on small local hospitals following large, competitive events despite safety measures taken at the venue. Such safety measures include on-site health and safety management, license applications, local authority liaisons, crowd management planning, fire safety management, crisis management planning, safe working practice documents, and pedestrian management solutions [4, 11].

Motocross involves travelling at high speeds up to 90mph [12]. The hazardous challenge and adrenaline surge appears to attract the young male racers. This study shows the impact and incidence of related accidents. Such accidents can be high velocity in nature, often resulting in serious injury, as illustrated by our study. It has been documented that between 1997 and 2006 the overall injury rate from motocross increased by 240 percent, and the rate of spine injury by almost 500 percent [13].

The patients involved in this study were largely managed in our unit as in-patients or within our fracture clinics.

Four patients were transferred by air ambulance to specialist neurosurgical units with significant head injuries and two patients were transferred to our neighbouring specialist spinal centre for urgent stabilization of cervical spine fracture dislocations. Another 2 patients required transfer to a specialist cardiothoracic centre following life-threatening haemo/pneumothorax.

The cost burden of these injuries is worthy of discussion. The estimated cost of an acute hospital bed in the UK is approximately £250 per day which, when added to the cost of surgical intervention, totals approximately £5,000 per day, plus the fees for patient follow-up [14, 15]. In addition there is also the cost of parents and guardians requiring compassionate leave from their workplace to look after their injured child which will inevitably have a follow-on cost to the government.

The limitations of our study are that we did not look at the size of the motorbike, rider experience, course design, and course safety provisions and therefore we cannot make any conclusions about their impact on safety. The patients included in this study were followed up until the present time, and include 5 patients who sustained significant disability requiring full-time care.

It is of interest that nearly all injuries in our series occurred at a formal track. A large number of injuries (65) were sustained from collisions or from racers being run over by other cyclists. Other authors have suggested that fewer riders on the course could reduce the frequency and severity of injuries [16].

There could be an initiative for safer course design, restrictions on participant age and limitations to vehicle speeds in an effort to help reduce the incidence and the severity of injury. Parents and coaches should consider these injuries and assess the benefits versus risks with regard to missed academic time and cost of medical treatment. It should also be noted that motocross may not be appropriate for some children if they do not have the maturity, coordination and dexterity to safely operate a motor vehicle [17]. These events, usually held at weekends, can also generate a sudden trauma workload often stretching local hospital facilities, at a time when resources are reduced. This can result in a consequential delay in the treatment of other patients, both acute and elective. It may also be beneficial for event organisers and the sport’s governing body (Auto Cycle Union Ltd) to review the protective equipment available and to collaborate with industry, in an effort to improve both safety and outcome, primarily for the competitor.

## Conclusion

The results from this study highlight the frequency and severity of motocross-related injuries in the paediatric population in the UK. This may assist in providing recommendations and guidelines to governing bodies and to parents. We would recommend that children <16 years of age should complete a mandatory education class with a formal assessment before being declared competent to race [18]. The injuries sustained during motocross have significant resource implications, especially for smaller rural hospitals, as shown by the number of injuries doubling in the last 4 years. Given the findings of this study, it is important that the receiving trauma teams when confronted with a motocross injury take into account the mechanisms and prepare for significant injuries resulting from this high-energy trauma.

This study outlines the inherent dangers of motocross in children and adolescents and the potential severe injuries which can occur as well as highlighting the significant resource implications for the health care system in treating these injuries and perhaps the long-term sequelae of some of the more devastating trauma.
