# Erratum to: An epidemiological study of paediatric motocross injuries in the United Kingdom

**DOI:** 10.1007/s11832-015-0702-8

**Published:** 2015-11-09

**Authors:** Rohit Singh, Akshay Malhotra, Nigel Kiely, Stuart Hay

**Affiliations:** Robert Jones Agnes Hunt Orthopaedic Hospital, Oswestry, UK; Royal Shrewsbury Hospital, Shrewsbury, Shropshire SY38XQ UK

## Erratum to: J Child Orthop DOI 10.1007/s11832-015-0685-5

In the original publication, the name of one of the co-authors has been published incorrectly. The correct name should be **Nigel Kiely**.

Furthermore, the affiliation of Dr. Rohit Singh and Dr. Stuart Hay is included now.

Royal Shrewsbury Hospital, Shrewsbury, Shropshire SY38XQ, UK.

